# Chemerin-induced mitochondrial dysfunction in skeletal muscle

**DOI:** 10.1111/jcmm.12487

**Published:** 2015-03-06

**Authors:** Qihai Xie, Yujie Deng, Chenglin Huang, Penghao Liu, Ying Yang, Weili Shen, Pingjin Gao

**Affiliations:** a State Key Laboratory of Medical Genomics, Shanghai Key Laboratory of Hypertension, Department of Hypertension Ruijin Hospital, Shanghai Jiaotong University School of MedicineShanghai, China; b Department of Endocrine and Metabolic Diseases, Shanghai Institute of Endocrine and Metabolic Diseases, Shanghai Clinical Center for Endocrine and Metabolic Diseases, Ruijin Hospital, Shanghai Jiaotong University School of MedicineShanghai, China

**Keywords:** chemerin, mitochondrial dysfunction, autophagy, skeletal muscle

## Abstract

Chemerin is a novel adipocyte-derived factor that induces insulin resistance in skeletal muscle. However, the effect of chemerin on skeletal muscle mitochondrial function has received little attention. In the present study, we investigated whether mitochondrial dysfunction is involved in the pathogenesis of chemerin-mediated insulin resistance. In this study, we used recombinant adenovirus to express murine chemerin in C57BL/6 mice. The mitochondrial function and structure were evaluated in isolated soleus muscles from mice. The oxidative mechanism of mitochondrial dysfunction in cultured C2C12 myotubes exposed to recombinant chemerin was analysed by western blotting, immunofluorescence and quantitative real-time polymerase chain reaction. The overexpression of chemerin in mice reduced the muscle mitochondrial content and increased mitochondrial autophagy, as determined by the increased conversion of LC3-I to LC3-II and higher expression levels of Beclin1 and autophagy-related protein-5 and 7. The chemerin treatment of C2C12 myotubes increased the generation of mitochondrial reactive oxygen species, concomitant with a reduced mitochondrial membrane potential and increased the occurrence of mitochondrial protein carbonyls and mitochondrial DNA deletions. Knockdown of the expression of chemokine-like receptor 1 or the use of mitochondria-targeting antioxidant Mito-TEMPO restored the mitochondrial dysfunction induced by chemerin. Furthermore, chemerin exposure in C2C12 myotubes not only reduced the insulin-stimulated phosphorylation of protein kinase B (AKT) but also dephosphorylated forkhead box O3α (FoxO3α). Chemerin-induced mitochondrial autophagy likely through an AKT-FoxO3α-dependent signalling pathway. These findings provide direct evidence that chemerin may play an important role in regulating mitochondrial remodelling and function in skeletal muscle.

## Introduction

Chemerin is a new adipokine associated with obesity and the metabolic syndrome 1, which is correlated with inflammatory cytokines, such as high-sensitivity C-reactive protein, interleukin-6 (IL-6) and tumour necrosis factor-α (TNF-α) 2. Previous studies have established that chemerin has proinflammatory chemoattractant properties elicited through interaction with the G protein-coupled receptor chemokine-like receptor 1 (CMKLR1) 3–5, which is expressed by macrophages, natural killer cells, immature dendritic cells, endothelial cells and skeletal muscle cells. CMKLR1-deficient mice are unresponsive to chemerin, which indicates that CMKLR1 is the physiological receptor for chemerin 6. Chemerin has been shown to impair insulin signalling and glucose uptake in skeletal muscle 7. However, chemerin treatment can enhance insulin signalling, potentates insulin-stimulated glucose uptake in 3T3-L1 adipocytes 8 and modulates adipogenesis 1. More recently, chemerin-knockout mice demonstrated increased glucose production in the liver. These data suggest that chemerin may play different roles in regulating muscle, adipose tissue and liver. Therefore, the role of chemerin in the development of insulin resistance and the regulation of energy metabolism has attracted more attention.

It has been indicated that the manner in which chemerin causes insulin resistance in skeletal muscle is linked to multiple mechanisms. Chemerin exposure has been found to reduce insulin-stimulated AKT phosphorylation, to activate extracellular signal-regulated kinases-1/2 and to increase proinflammatory cytokines, such as TNF-α and IL-6, which could impair insulin action 7. The inhibition of insulin signalling by chemerin at multiple levels, including insulin receptor substrate 1 and glycogen synthase kinase 3 phosphorylation, has been demonstrated in primary human skeletal muscle cells 7. These findings indicate that various mechanisms are likely to be involved in chemerin-induced insulin resistance. However, the effect of chemerin on skeletal muscle mitochondrial function has received little attention.

The mitochondria play important roles in biosynthetic pathways, cellular energetics, cellular redox homoeostasis, signalling, calcium buffering and the regulation of programmed cell death 9. Mitochondrial quality control is accomplished by the dynamic interplay of fusion, fission, autophagy and mitochondrial biogenesis 10. Proteins that control fusion include mitofusin-1 (MFN1), Mfn2 and optic atropy-1 (OPA-1). Fission is regulated by dynamic related protein-1 (DRP-1) and fission-1 (FIS-1) 11. The mitochondrial network dynamics are sensitive to various physiological and pathological stimuli, and alterations in the cellular energy status associated with the development of insulin resistance can also be achieved through mitochondrial network dynamics 12. Our previous study demonstrated that mitochondrial loss is caused by autophagy in the skeletal muscle of diabetic Goto-Kakizaki (GK) rats and that amelioration of excessive muscle autophagy in GK rats can improve insulin sensitivity 13. Therefore, mitochondrial biogenesis and fragmentation may act as regulators of insulin resistance and muscle protein degradation 14.

However, the effects of chemerin on the muscle mitochondrial content and its subsequent influence on muscle function have not yet been elucidated. In this study, we showed that mitochondrial oxidative stress is involved in the regulation of the mitochondrial content and the function of cultured C2C12 myotubes by altering the expression levels of genes related to mitochondrial biogenesis and the antioxidant system. We therefore focused on the effects of chemerin on muscle mitochondria, particularly on the regulation of mitochondrial remodelling, and their relationship with the pathophysiology of insulin resistance in skeletal muscle.

## Materials and methods

See Data S1 for further details.

## Results

### Effect of chemerin on the mitochondrial dynamic in skeletal muscle

**Figure 1:**
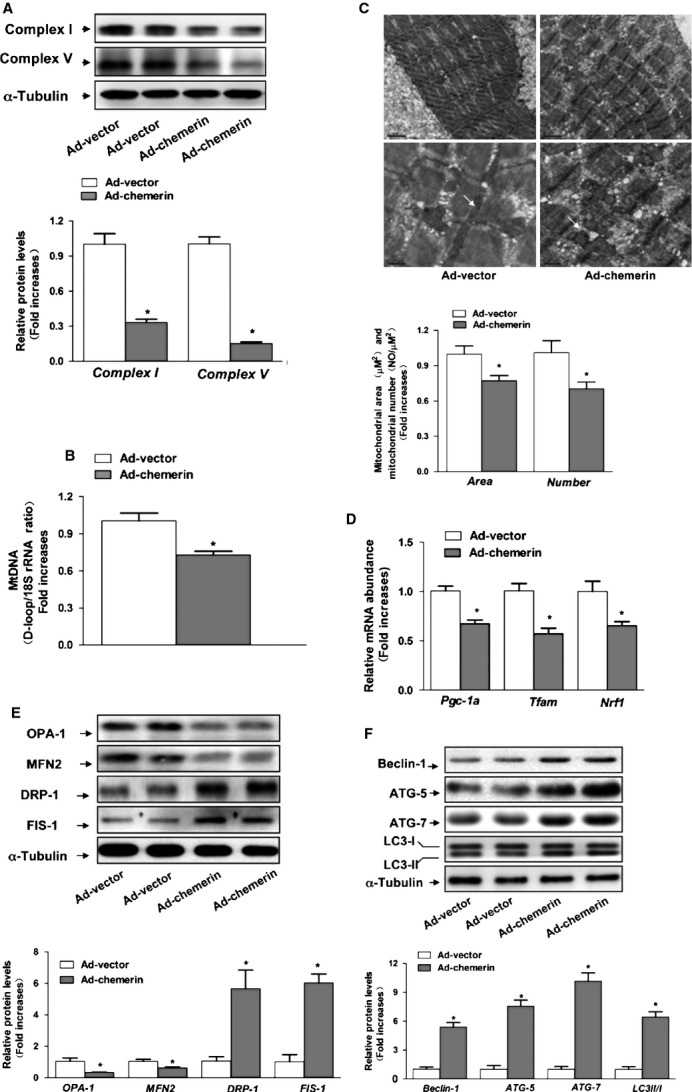
Effect of chemerin on the mitochondrial dynamics in skeletal muscle. (A) Expression of mitochondrial complexes I and V. (B) The mtDNA contents were determined by real-time PCR. (C) Morphometric analyses of the surface area of the mitochondria and mitochondrial density were performed. (D) mRNA expression of *Pgc-1a*, *Nrf1* and *Tfam*. (E) Western blot images and quantitative analyses of OPA1, MFN2, DRP1and FIS-1. Upper: representative western blot image; bottom: quantitative analyses of the bands determined by densitometry. (F) Western blots images and quantitative analyses of ATG5, ATG7, Beclin-1 and LC3B. The results are expressed as fold increases over the control. The data are presented as the means ± SEM (*n* = 12). *P < 0.05 *versus* the control group.

C57BL/6 mice were injected with 1 × 10^9^ particles of Ad-chemerin or Ad-vector alone to examine the effect of chemerin overexpression *in vivo*. One week after administration, the injection of Ad-chemerin exhibited sustained higher serum chemerin levels compared with Ad-vector (Figure S1). We investigated whether chemerin expression was accompanied by reduced mitochondrial content. As shown in Figure1A, the protein levels of mitochondrial respiratory complexes I and V were significantly lower in chemerin-expressing mice than in control mice. Similarly, the mitochondrial DNA copy number was similarly reduced in chemerin-expressing mice (Fig.1B). Furthermore, we measured the mitochondrial mass and density in chemerin-expressing and control mice by morphometric analysis at the ultrastructural level. In the soleus muscle, the mitochondrial area and density were markedly reduced in chemerin-expressing mice compared with the controls (Fig.1C). The levels of *Pgc-1*α, *Nrf1* and *Tfam* mRNA were markedly reduced in the soleus muscle of chemerin-expressing mice (Fig.1D). In addition, we found that chemerin significantly increased the expression of the mitochondrial fission-related proteins DRP1 and FIS-1 and decreased the mitochondrial fusion-related proteins MFN1 and OPA1 (Fig.1E). The conversion of LC3-I to LC3-II, an indicator of autophagy activation, was assessed by immunoblot analysis using LC3 antibody. The LC3-II abundance was increased by chemerin overexpression. We performed immunoblotting to detect the levels of the autophagy-associated proteins Beclin-1, ATG5 and ATG7. As expected, the autophagy-related proteins ATG5, ATG7 and Beclin-1 was markedly increased in chemerin-expressing mice (Fig.1F).

### Mitochondrial dysfunction in the skeletal muscle of chemerin-expressing mice

**Figure 2:**
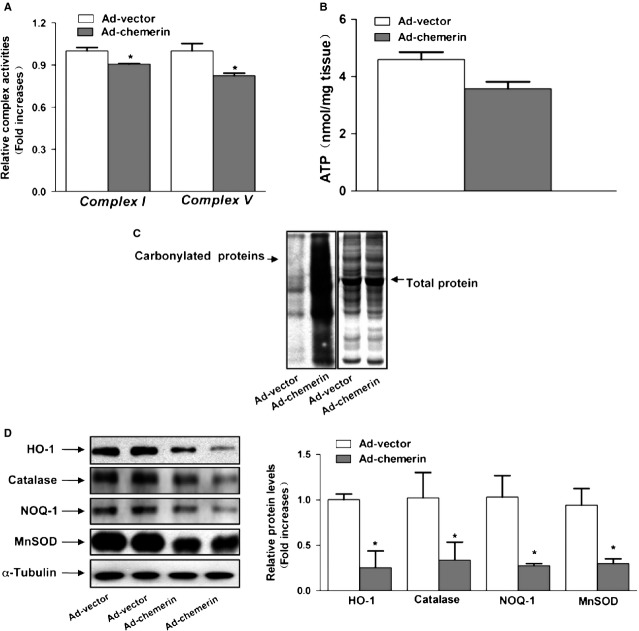
Mitochondrial dysfunction in the skeletal muscle of chemerin-expressing mice. (A) The activities of mitochondrial complexes I and V were assayed by a spectrometric assay. (B) ATP content in the soleus muscle. (C) The skeletal muscle protein carbonyls were detected by western blotting. Other polyacrylamide resolving gels loaded with the same quantity of the samples and stained with Coomassie Brilliant Blue R250 served as the loading control. (D) Western blots images and quantitative analyses of HO-1, catalase, NQO-1 and MnSOD. Left: representative western blot image; right: quantitative analyses of the bands determined by densitometry. The results are expressed as fold increases over the control. The values are presented as the means ± SEM. *P < 0.05 *versus* the control group.

We then measured the complex enzyme activity in skeletal muscle. Our measurement showed that the activity of mitochondrial complexes I and V were significantly lower in chemerin-expressing mice than in control mice (Fig.2A). The results indicated that decreased mitochondrial complex enzyme activity was accompanied by impairment of the mitochondrial protein levels in chemerin-expressing mice. We then investigated whether the decreased mitochondrial content was associated with a decrease in ATP synthesis. The ATP level of the muscle in chemerin-expressing mice exhibited a decreasing trend compared with that found in control mice; however, this difference was not statistically significant (Fig.2B).

### Increased oxidative stress in the skeletal muscle of chemerin-expressing mice

To test whether oxidative stress is increased in chemerin mitochondrial dysfunction, we investigated the muscular protein carbonylation level. The muscular protein carbonylation level was elevated in chemerin-expressing mice compared with Ad-vector mice (Fig.2C). The analysis of the antioxidant system showed that the protein levels of phase 2 enzymes (NQO-1 and HO-1) and the mitochondrially located antioxidant MnSOD were significantly decreased (Fig.2D). Taken together, these data suggest that oxidative stress in skeletal muscle may be a determinant of mitochondrial alterations in chemerin-expressing mice.

### Reduction of CMKLR1 reduces chemerin-induced mitochondrial dysfunction in C2C12 myotubes

**Figure 3:**
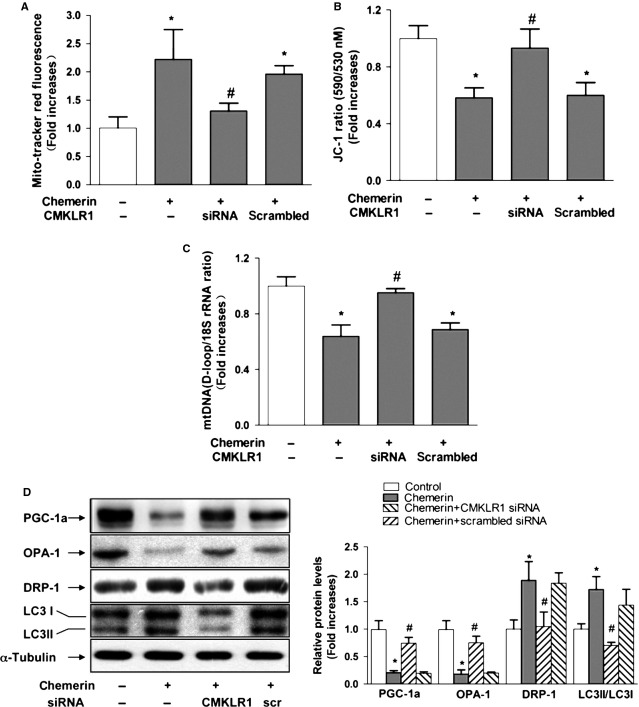
Reduction of CMKLR1 reduces chemerin-induced mitochondrial loss, mitochondrial oxidative capacity and autophagy in C2C12 myotubes. Forty-eight hours after transfection with siRNA, the cells were treated with chemerin for 4 24 hrs. (A) Quantification of the ROS levels of C2C12 myotubes. (B) The membrane potential of C2C12 myotubes with different treatments was quantified. (C) The mitochondrial DNA contents were determined by real-time PCR. (D) Left: representative western blot image; right: quantification of the protein expression levels of PGC-1α, OPA-1, DRP-1 and LC3B. The results are presented relative to the values in the control cells. The data are presented as the means ± SEM from four independent experiments. *P < 0.05 *versus* the control group; #*P* < 0.05 *versus* the chemerin group.

To determine the receptor responsible for the effects of chemerin on mitochondria, C2C12 cells were subjected to genetic blockade of CKMLR1 by siRNA. We confirmed that CKMLR1 expression was decreased by 80% after transfection with CKMLR1 siRNA (data not shown). The knockdown of CMKLR1 receptor expression completely reversed the decrease in the mitochondrial DNA content, whereas the increase in Reactive oxygen species and the decrease in MMP induced by chemerin (0.5 μg/ml) were significantly suppressed by blockade of CKMLR1 (Fig.3A–C). We also examined the expression levels of genes involved in mitochondrial biogenesis (PGC1α and OPA1) and found that both PGC1α and OPA1 were decreased in the chemerin-treated group at 0.5 μg/ml. Consistent with its effects on the mitochondrial content, blockade by CKMLR1 siRNA completely reversed the chemerin-induced decrease in PGC1α and OPA1. Furthermore, the effect of chemerin on the LC3II/LC3I level in cells in which CKMLR1 was knocked down by siRNA was clearly reduced compared with its effects on cells transfected with the scrambled siRNA (control). Taken together, these data provide strong evidence that the treatment of C2C12 myotubes with chemerin triggers mitochondrial dysfunction through a mechanism that involves CMKLR1 (Fig.3D).

### Chemerin activated the AKT-FoxO3α signalling pathway in a CMKLR1-dependent manner

**Figure 4:**
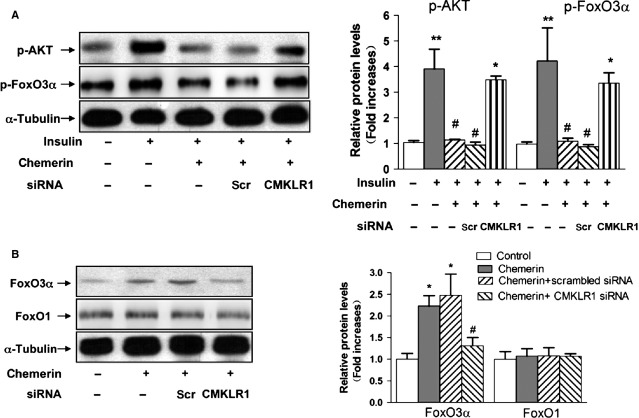
Chemerin activated the AKT-FoxO3α signalling pathway in a CMKLR1-dependent manner. (A) Forty-eight hours after transfection with CMKLR1 siRNA, the cells were pre-treated with or without chemerin (0.5 μg/ml) for 24 hrs, stimulated with insulin (100 nmol/l) for 15 min., solubilized in SDS sample buffer and analysed by western blotting with antibodies against p-AKT and p-FoxO3α. (Β) After the transfection of CMKLR1 siRNA, the cells were solubilized in SDS sample buffer and analysed by western blotting with antibodies against FoxO1 and FoxO3α. Left: representative western blot image; right: quantification of the protein expression. The results are presented relative to the values in the control cells. The data are presented as the means ± SEM from four independent experiments. *P < 0.05 *versus* the control group; #*P* < 0.05 *versus* the chemerin group.

Consistent with other studies, after incubation with chemerin, C2C12 myotubes showed a marked decrease in insulin-stimulated AKT phosphorylation at Ser473. Moreover, new evidence has revealed that increased mitochondrial fragmentation because of fission is required by the AKT-FoxO3α signalling axis 15. We therefore explored whether FoxO3α is involved in chemerin-induced autophagy in C2C12 myotubes. Insulin-induced FoxO3α activation was inhibited by pre-incubation with chemerin (0.5 μg/ml). The knockdown of CMKLR1 by siRNA significantly reversed the alterations in p-AKT and p-FoxO3α induced by chemerin. Consistent with the inhibition of p-FoxO3α, we detected elevated levels of FoxO3α in C2C12 myotubes upon treatment with chemerin, and this effect was attenuated by the knockdown of CMKLR1 expression. The total protein expression levels of FoxO1 were unchanged (Fig.4A and B).

### Mitochondrial ROS mediate chemerin-induced autophagy pathway

**Figure 5:**
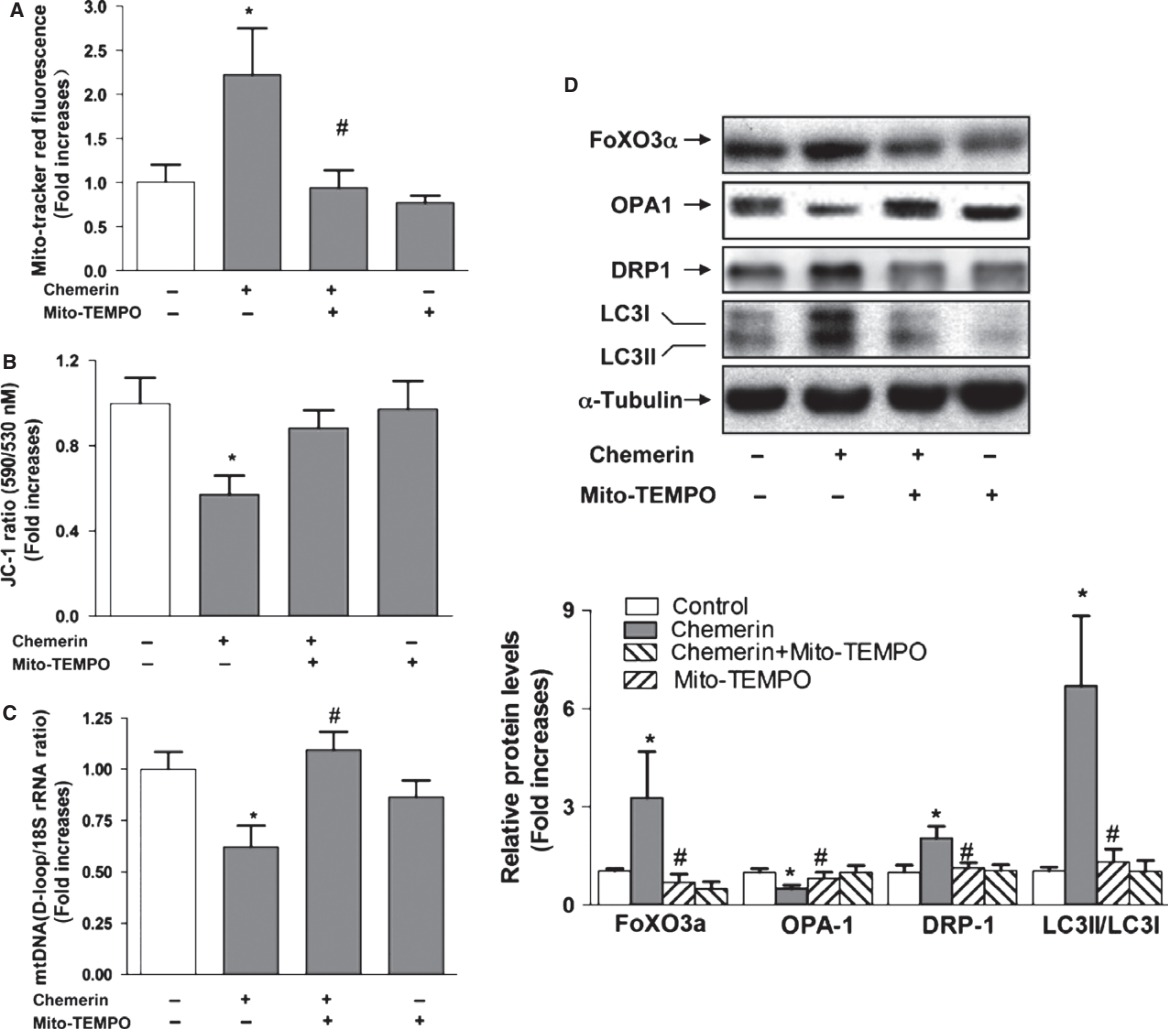
Mitochondrial ROS mediate the chemerin-induced autophagy pathway. (A) C2C12 myotubes were pre-incubated with Mito-TEMPO (10 μM) for 1 hr prior to treatment with chemerin for 4 hrs. The ROS levels of C2C12 myotubes were quantified. (B) C2C12 myotubes were pre-incubated with Mito-TEMPO (10 μM) for 1 hr and then treated with chemerin for 24 hrs. The membrane potential of the C2C12 myotubes was quantified. (C) The mitochondrial DNA contents were determined by real-time PCR. (D) After treatment, the cells were subsequently solubilized in SDS sample buffer and analysed by western blotting with antibodies against Foxo3α and LC3B. Upper: representative western blot image; bottom: quantification of the protein expression levels of FoxO3α, OPA-1, DRP1 and LC3B. The final results are presented as fold increases over the control. The data are presented as the means ± SEM from four independent experiments. *P < 0.05 *versus* the control group; #*P* < 0.05 *versus* the chemerin group.

Mitochondrial ROS have been implicated in autophagy 16,17 and apoptosis 18. In this study, we determined whether ROS mediate chemerin-induced mitochondrial dysfunction. As shown in Figure5A, the C2C12 myotubes were pre-incubated with 10 μM Mito-TEMPO for 1 hr prior to a 4-hr treatment with 0.5 μg/ml chemerin, and the mitochondrial ROS production was then measured with MitoTracker Red. The mitochondria-targeted antioxidant Mito-TEMPO not only suppressed the basal level of ROS generation but also significantly suppressed the chemerin-mediated ROS production. Furthermore, pre-treatment with Mito-TEMPO reversed the decrease in mtDNA content and the decrease in MMP induced by chemerin (Fig.5B and C). The expression levels of FoxO3α and DRP-1 and the conversion of LC3-I to LC3-II induced by chemerin treatment were significantly reduced by the presence of Mito-TEMPO, whereas OPA-1 was increased (Fig.5D). Taken together, these results suggest that oxidative stress induced by chemerin triggers alteration of the mitochondrial dynamics and that the antioxidant Mito-TEMPO may mitigate oxidative stress and reverse the mitochondrial dynamics.

### FoxO3α signalling is required for chemerin-induced autophagy

**Figure 6:**
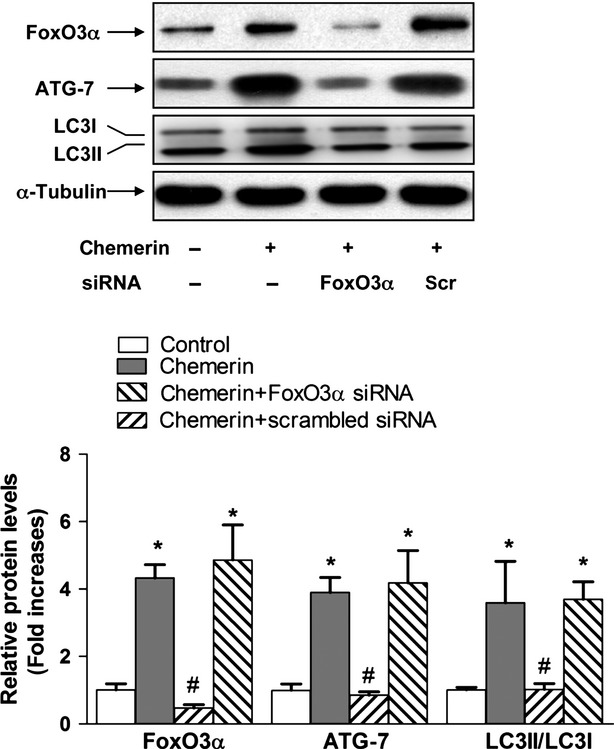
FoxO3α signalling is required for chemerin-induced autophagy. C2C12 myotubes were transfected with FoxO3α siRNA or scrambled siRNA. Top: representative western blot images of FoxO3α, ATG7 and LC3B; bottom: quantification of protein expression. The final results are presented as fold increases over the control. The data are presented as the means ± SEM from four independent experiments. *P < 0.05 *versus* the control group; #*P* < 0.05 *versus* the chemerin group.

We tested whether the knockdown of FoxO3α can influence C2C12 myotube autophagy. The RNAi construct of Foxo3a but not its scramble form was able to suppress FoxO3α expression (Fig.6). The knockdown of FoxO3α could attenuate the increases in ATG7 and LC3B conversion induced by chemerin in C2C12 myotubes. These data suggest that chemerin induces mitochondrial autophagy likely through a FoxO3α-dependent signalling pathway.

## Discussion

In the present study, we observed that chemerin reduced the mitochondrial content in cultured myotubular cells and the skeletal muscle of mice. The treatment of C2C12 myotubes with chemerin increased the generation of mitochondrial ROS concurrent with the conversion of LC3B-I to LC3B-II and the up-regulation of the autophagy-related genes Beclin-1, ATG 7 and ATG 5. These findings imply that chemerin causes alterations in the mitochondrial function and dynamics, which have been implicated in the regulation of insulin resistance in skeletal muscle.

To determine the receptor responsible for the effects of chemerin on the mitochondrial content, we used RNA interference of the receptor in C2C12 myotubes. We found that the decrease in mitochondrial content induced by chemerin could be reversed by the genetic blockade of CMKLR1. The change in the muscle mitochondrial content was paralleled by the mitochondrial protein levels of complex I and complex V. Consistent with this finding, chemerin decreased the expression levels of *Pgc1*α and *Tfam*, which positively regulate mitochondrial biogenesis, in a CMKLR1-dependent manner. In addition, we showed that the ablation of CMKLR1 decreased the chemerin-induced expression of Beclin-1 and LC3II conversation. Furthermore, we observed a marked increase in the levels of DRP-1 and Fis-1 in chemerin-expressing mice. These results suggest that CMKLR1 may be the primary receptor for mediating the chemerin-induced alterations in mitochondrial dynamics in C2C12 myotubes.

Mitochondrial ROS may play an important role in mediating mitochondrial dynamics. In fact, because of their proximity to ROS, mitochondrial proteins, lipids and DNA are believed to be the primary targets of oxidative damage during stress, creating a mitochondrial free radical ‘vicious cycle’ of injury 19. In the present study, we found that protein carbonyls were significantly elevated in the skeletal muscle of chemerin-expressing mice. In addition, mitochondria-specific MnSOD and phase 2 enzymes (NQO1, HO-1) decreased in a corresponding manner. Consistent with the *in vivo* results, we found that chemerin increased mitochondrial ROS production in C2C12 myotubes in a concentration-dependent manner and that the mitochondrial membrane potential was lowered by chemerin, which was associated with an increase in mitochondrial ROS. The silencing of CMKLR1 expression restored mitochondrial dysfunction (*i.e*. mitochondrial ROS, MMP and mitochondrial mass) in C2C12 myotubes. These effects were similar to those observed with the mitochondrial-targeted antioxidant Mito-TEMPO. The findings suggest that the CMKLR1-dependent pathway augments ROS production, which is known to promote autophagy and reduce the mitochondrial content in skeletal muscle.

Because we found that the inhibition of ROS production improved the mitochondrial content and decreased autophagy, ROS may influence the chemerin signalling pathway. Consistent with other studies, we found that chemerin reduced insulin-stimulated AKT phosphorylation. In addition, chemerin reduced insulin-stimulated FoxO3α phosphorylation in a manner dependent on CMKLR1. FoxO3α is a downstream target of the phosphatidylinositol-3 kinase (PI3K)/AKT pathway. In the current study, we detected elevated levels of FoxO3α in C2C12 myotubes upon treatment with chemerin, and this effect was attenuated by the knockdown of CMKLR1 expression. The total protein expression levels of FoxO1 were unchanged. siRNA specific to FoxO3α blocked the enhancement of autophagy and a loss of mitochondria in C2C12 myotubes treated with chemerin. These results suggest that an enhanced activity of FoxO3α may be responsible for the autophagic process.

Although decreased mitochondrial function and activity in skeletal muscle have been documented in obesity and 2 type diabetes, the involvement of the mitochondrial dynamics in the pathogenesis of metabolic disorders has been given little attention. The current knowledge is that aberrant mitochondrial fission is causally associated with mitochondrial dysfunction and insulin resistance in skeletal muscle 20. It has been suggested that the mitochondrial dynamics form a bridge between mitochondrial dysfunction and insulin resistance 21. We provide evidence that chemerin-induced mitochondrial dysfunction is involved in decreased mitochondrial biogenesis and enhanced mitochondrial autophagy.

Because the mitochondrial electron transport chain is considered the major source of ROS in tissue, a certain level of ROS is required for controlling the mitochondrial quality. Chemerin may cause an imbalance between the generation and removal of ROS. Excessive ROS, in turn, trigger the activation of autophagy and mitochondrial fission and loss, and finely regulate the tightly controlled balance between fusion and fission processes. Insulin-like growth factor I and insulin have been shown to promote the net protein accumulation of mature myotubes and adult muscle fibres. Insulin resistance is a characteristic feature of many systemic diseases and appears to contribute to muscle wasting. Our results indicate that chemerin can disrupt insulin signalling in muscle, potentially contributing to its wasting effect.

In conclusion, we provide evidence that mitochondrial dynamics occur in the skeletal muscle of animals and in cultured muscle cells in response to high levels of chemerin. The inhibition of mitochondrial ROS production protected muscle cells against mitochondrial dysfunction. Thus, our results establish a causative link between mitochondrial dynamics and metabolic deterioration and imply that the disruption of mitochondrial dynamics in skeletal muscle may underlie the pathogenesis of insulin resistance. Finally, regulation of the mitochondrial morphology may provide a novel therapeutic strategy for insulin resistance and type 2 diabetes.

## Supporting Information

**Figure S1** Western blot analysis of chemerin expression in serum samples of C57BL/6 mice 1 week after injection with Ad-chemerin or Ad-vector.

## References

[b1] Roh SG, Song SH, Choi KC (2007). Chemerin–a new adipokine that modulates adipogenesis *via* its own receptor. Biochem Biophys Res Commun.

[b2] Chu SH, Lee MK, Ahn KY (2012). Chemerin and adiponectin contribute reciprocally to metabolic syndrome. PLoS ONE.

[b3] Zabel BA, Silverio AM, Butcher EC (2005). Chemokine-like receptor 1 expression and chemerin-directed chemotaxis distinguish plasmacytoid from myeloid dendritic cells in human blood. J Immunol.

[b4] Wittamer V, Franssen JD, Vulcano M (2003). Specific recruitment of antigen-presenting cells by chemerin, a novel processed ligand from human inflammatory fluids. J Exp Med.

[b5] Berg V, Sveinbjornsson B, Bendiksen S (2010). Human articular chondrocytes express ChemR23 and chemerin; ChemR23 promotes inflammatory signalling upon binding the ligand chemerin(21-157). Arthritis Res Ther.

[b6] Luangsay S, Wittamer V, Bondue B (2009). Mouse ChemR23 is expressed in dendritic cell subsets and macrophages, and mediates an anti-inflammatory activity of chemerin in a lung disease model. J Immunol.

[b7] Sell H, Laurencikiene J, Taube A (2009). Chemerin is a novel adipocyte-derived factor inducing insulin resistance in primary human skeletal muscle cells. Diabetes.

[b8] Takahashi M, Takahashi Y, Takahashi K (2008). Chemerin enhances insulin signaling and potentiates insulin-stimulated glucose uptake in 3T3-L1 adipocytes. FEBS Lett.

[b9] Ungvari Z, Sonntag WE, Csiszar A (2010). Mitochondria and aging in the vascular system. J Mol Med.

[b10] Carreira RS, Lee P, Gottlieb RA (2011). Mitochondrial therapeutics for cardioprotection. Curr Pharm Des.

[b11] Manczak M, Calkins MJ, Reddy PH (2010). Impaired mitochondrial dynamics and abnormal interaction of amyloid beta with mitochondrial protein Drp1 in neurons from patients with Alzheimer's disease: implications for neuronal damage. Hum Mol Genet.

[b12] Bo H, Zhang Y, Ji LL (2010). Redefining the role of mitochondria in exercise: a dynamic remodeling. Ann N Y Acad Sci.

[b13] Yan J, Feng Z, Liu J (2012). Enhanced autophagy plays a cardinal role in mitochondrial dysfunction in type 2 diabetic Goto-Kakizaki (GK) rats: ameliorating effects of (-)-epigallocatechin-3-gallate. J Nutr Biochem.

[b14] Civitarese AE, Ravussin E (2008). Mitochondrial energetics and insulin resistance. Endocrinology.

[b15] Powers SK, Wiggs MP, Duarte J (2012). Mitochondrial signaling contributes to disuse muscle atrophy. Am J Physiol Endocrinol Metab.

[b16] Qian W, Liu J, Jin J (2007). Arsenic trioxide induces not only apoptosis but also autophagic cell death in leukemia cell lines *via* up-regulation of Beclin-1. Leuk Res.

[b17] Yu L, Wan F, Dutta S (2006). Autophagic programmed cell death by selective catalase degradation. Proc Natl Acad Sci USA.

[b18] Pelicano H, Carney D, Huang P (2004). ROS stress in cancer cells and therapeutic implications. Drug Resist Updat.

[b19] Balaban RS, Nemoto S, Finkel T (2005). Mitochondria, oxidants, and aging. Cell.

[b20] Jheng HF, Tsai PJ, Guo SM (2012). Mitochondrial fission contributes to mitochondrial dysfunction and insulin resistance in skeletal muscle. Mol Cell Biol.

[b21] Zorzano A, Liesa M, Palacin M (2009). Mitochondrial dynamics as a bridge between mitochondrial dysfunction and insulin resistance. Arch Physiol Biochem.

