# Supplementary material for: Chemerin-induced mitochondrial dysfunction in skeletal muscle
Source: J Cell Mol Med. 2015 Mar 6;19(5):986–95. doi: 10.1111/jcmm.12487 (PMC4420601; doi:10.1111/jcmm.12487)
Supplement: Supplementary file 1 [file jcmm0019-0986-sd1.doc]

**Materials and methods**

***Materials***

Mito-Tracker Green FM, Mito-Tracker Red CM-H2XRos, and tetraethylbenzimidazolecarbocyanine iodide (JC-1) were purchased from Molecular Probes (Eugene, OR, USA). Anti-LC3B (2775), anti-ATG5 (12994) and anti-Beclin-1 (3738) were obtained from Cell Signaling Technology (Beverly, MA, USA). Antibodies against PGC-1α (sc-13067), MFN1 (sc-50330), MFN2 (sc-50331), OPA-1 (sc-367890), DRP-1 (sc-32898) and MnSOD (sc-18504) were purchased from Santa Cruz Biotechnology (Santa Cruz, CA, USA). The FL-AA bioluminescent assay kit and antibodies against α-tubulin (T6199), and ATG7 (A2856) were purchased from Sigma (St. Louis, MO, USA). Recombinant mouse chemerin and anti-chemerin (MAB2325) were obtained from R&D Systems (Minneapolis, MN, USA). Peroxidase-conjugated rabbit anti-goat IgG (305-035-003), goat anti-mouse IgG (115-035-003), and goat anti-rabbit IgG (111-035-003) were obtained from Jackson ImmunoResearch (West Grove, USA). The CMKLR1-siRNA (sc-44634), control-siRNA (sc-37007), and Mito-TEMPO ([sc-221945](http://www.scbt.com/search/redirect.php?location=datasheet-221945-mito-tempo.html&searchPhrase=mito-tempo&datasheet=sc-221945&tableName=&productType=&page=1)) were purchased from Santa Cruz (Santa Cruz, CA, USA). SignalSilence® FoxO3α siRNA I (Mouse Specific) (8620) and SignalSilence® Control siRNA (6568) were purchased from Cell Signaling Technology (Beverly, MA, USA). The Reverse Transcription System kit and SYBR Green were obtained from Promega (Manheim, Germany). HotStarTaq was produced by TaKaRa (Otsu, Shiga, Japan). The *Pgc1α*, *18S rRNA*, *Nrf1*, *Tfam* and *D-loop* primers were synthesized by Bioasia Biotech (Shanghai, China).

***Construction of recombinant adenovirus vector containing the chemerin gene***

Briefly, full-length mouse chemerin (accession number NM_027852) was amplified with the following primers: forward, 5'-ATCGGTCGACGCATGAAGTGCTTGCTGATCTC-3'; and reverse, 5'-ATCGCTCGAGTTTGGTTCTCAGGGC-3'. The PCR product was cloned into the pGEM-T easy vector and confirmed by DNA sequencing. The confirmed chemerin cDNA was cloned between the SalⅠ and XhoⅡ sites of the pShuttle-IRES-hrGFP-1 vector with the green fluorescent protein gene (GFP), and the vector was linearized by PmeI digestion and recombined with pAdEasy-1 in BJ5183 *Escherichia coli.* After confirmation of recombination by Pac-1 restriction digestion and sequencing, the Pac1-linearized recombinant Ad plasmids were then transfected into AD293 cells using Lipofectamine 2000. The virus was harvested, tittered, and stocked at a concentration of 1×10^9^ plaque forming units/ml.

*Animal studies*

Six-week-old male C57BL/6 mice were purchased from SLAC Laboratory Animal Co. Ltd. (Shanghai, China). All of the animals were housed at 23±2ºC with 12-h light/12-h dark cycles and given access to food and water *ad libitum*. The experiments were performed in accordance with the Guidelines for Animal Experiments of the Institute for Nutritional Sciences of the Chinese Academy of Sciences. All of the experiments involving animals were conducted in conformance with the Guide for the Care and Use of Laboratory Animals published by the US National Institutes of Health (NIH Publication, 8th edition, 2011). A total of 30 mice were randomly divided into two groups of 15 mice each. The mice were injected intravenously via the tail vein with 1 × 10^9^ particles of Ad-chemerin or Ad-vector. After one week, the animals were anesthetized with an intraperitoneal injection of sodium pentobarbital (60 mg/kg) and were sacrificed to obtain soleus muscle tissue.

***Chemerin expression***

Murine serum samples were subjected to 15% (w/v) SDS-PAGE, and the proteins were then transferred to nitrocellulose membranes. Murine chemerin was detected with rat anti-mouse chemerin antibody (1:1,000). The membranes were washed three times with TBST and then incubated with peroxidase-conjugated secondary antibody for 1 h at room temperature. Western blots were developed using chemoluminescence and quantified by scanning densitometry .

***Assays for mitochondrial enzyme activities***

The NADH-CoQ oxidoreductase (complex I) activity was assayed by monitoring the reduction of 2,6-dichlorophenolindophenol (DCPIP) at 600 nm upon the addition of assay buffer (10× buffer containing 0.5 mol/l Tris-HCl, pH 8.1, 1% BSA, 10 μmol/l antimycin A, 2 mmol/l NaN_3_, and 0.5 mmol/l coenzyme Q1) . The final concentration of mitochondrial protein was 25 μg/ml. The reaction was started by the addition of 200 μmol/l NADH and was scanned at 600 nm for 2 min. Rotenone (3 μmol/l) was added to the reaction system as a blank control. The complex V activity was measured as the oligomycin-sensitive, Mg^2+^-ATPase activity . The process was performed by measuring the increase in NADPH at 340 nm upon the addition of 10 mmol/l HEPES (pH 8.0), 20 mmol/l succinate, 20 mmol/l glucose, 3 mmol/l MgCl_2_, 11 mmol/l AMP, 0.75 mmol/l NADP^+^, 10 mmol/l K_2_HPO_4_, 4 U/ml hexokinase, 2 U/ml glucose-6-phosphate dehydrogenase, and 60 μg/ml mitochondria . The reaction was initiated by the addition of 1 mmol/l ADP. All of the assays were performed at 30ºC.

***Measurement of muscle ATP content***

Frozen muscle biopsies were crushed and homogenized in 2 ml of cold trichloroacetic acid (2.5% vol/vol). The homogenate was centrifuged for 10 min at 1,000 × g and 4°C. The supernatant was neutralized with 1 mol/l Tris base. The resultant supernatant was assayed for ATP content using the FL-AA bioluminescent assay kit. The pellet was neutralized with 1 ml of 0.5 mol/l NaOH, and the protein content was determined by the Bradford method. The muscle content of ATP is expressed as micromoles per gram of protein .

***Transmission electron microscopy***

A portion of fresh soleus was fixed in 2.5% (v/v) glutaraldehyde in phosphate buffered saline, post-fixed in 4% (w/v) osmium tetroxide, and embedded in Epon resin. Ultrathin sections (50- to 80-nm-thick) were prepared, stained with lead citrate and uranyl acetate, and observed with a transmission electron microscope (CM 10; Philips, Eindhoven, the Netherlands). Statistical analyses of the cross-sectional area of the mitochondria and mitochondrial densities were performed using software Image-Plo Plus v6.0.

***C2C12 cell culture and differentiation***

Mouse C2C12 myoblasts were purchased from the ATCC (Manassas, VA, USA) and maintained in Dulbecco’s modified Eagle medium (DMEM) supplemented with 10% foetal bovine serum at a confluence of 60% to 70%. To initiate differentiation, the cells were allowed to reach 100% confluence, and the medium was changed to DMEM containing 2% horse serum and freshly replaced every two days. Full differentiation with myotube fusion and spontaneous twitching were observed at 6 to 8 days.

*Transfection of siRNA*

On day 6 after C2C12 myotube differentiation, the media of the C2C12 cells in six-well plates were changed to siRNA transfection medium, and the cells were transfected with the following siRNAs: (1) CMKLR1-siRNA (100 nmol/l) or control-siRNA (100 nmol/l); (2) FoxO3-siRNA (100 nmol/l) or SignalSilence® Control siRNA (100 nmol/l). Briefly, siRNA (100 nM) was formulated with the siRNA transfection reagent (Santa Cruz Biotechnology) according to the instructions provided by the manufacturer. The transfection complex was diluted in siRNA transfection medium (Santa Cruz Biotechnology) and added directly to the cells. After 24 h of transfection with siRNA, C2C12 myotubes were used for the subsequent experiments. The depletion of endogenous CMKLR1 or FoxO3α by siRNA was confirmed by western blot.

***Mitochondrial mass***

A fluorescent probe (Mito-Tracker Green FM) was used to determine the mitochondrial mass of the C2C12 myotubes. After treatment, the C2C12 myotubes were trypsinized and then incubated with 100 nmol/l MitoTracker Green FM in KRH buffer for 30 min at 37°C. The cells were centrifuged at 3,000 × *g* and 4°C for 5 min and resuspended in 400 μl of fresh KRH buffer. The fluorescence was analysed using a fluorescence spectrometer at an excitation wavelength of 490 nm and an emission wavelength of 516 nm. The optical density values were normalized to the protein levels .

*Mitochondrial reactive oxygen species measurement*

After treatment, the C2C12 myotubes were incubated with 100 nmol/l MitoTracker Red for 30 min at 37°C. At the end of the incubation period, the cells were washed three times with cold PBS and then lysed with 0.5% Triton X-100. The cell lysates were centrifuged at 1500 × g for 10 min, and the supernatants were measured with a fluorescence spectrometer at an excitation wavelength of 515 nm and an emission wavelength of 535 nm. The optical density values were normalized to the protein levels .

*Mitochondrial membrane potential assessment*

The mitochondrial membrane potential (MMP) is sensitive to toxic insults and is used for measuring both cell viability and mitochondrial function. JC-1 was employed to quantitatively determine alterations in the MMP. Mitochondrial depolarization was indicated by a decrease in the red-to-green fluorescence intensity ratio. Briefly, C2C12 myotubes were cultured in a 96-well plate with different treatments, incubated with 10 μg/ml JC-1 for 30 min at 37°C, and scanned with a fluorescence spectrometer. The fluorescence ratio (590 to 530 nm) was used for quantitative analysis .

*Cell respiration*

After treatment, the cells were washed in Krebs-Ringer-HEPES buffer plus 0.1% (w/v) BSA. The cells from each condition were divided into triplicate aliquots in a BD Oxygen Biosensor System plate. The plates were sealed and read using a fluorescence spectrometer at 1-min intervals for 60 minutes at an excitation wavelength of 485 nm and an emission wavelength of 630 nm. The results are expressed as the slope of the fluorescence intensity using the control V_max_ as 1 .

***Western blot***

The soluble lysates (10 μg per lane) were subjected to 8%, 10%, or 12% (w/v) SDS-PAGE, and the proteins were then transferred to nitrocellulose membranes and blocked with 5% (w/v) non-fat milk/Tris-buffered saline Tween 20 (TBST) for 1 h at room temperature. The membranes were incubated overnight at 4°C with primary antibodies directed against α-tubulin (1:5000), anti-LC3B (1:1000), anti-ATG5-12 (1:1000), anti-ATG7 (1:1000), anti-Beclin-1 (1:1000), anti-p-AKT (1:1000), anti-p-FoxO3α (1:1000), and anti-FoxO3α (1:1000) in 5% (w/v) milk/TBST. The membranes were washed three times with TBST and then incubated with peroxidase-conjugated secondary antibody for 1 h at room temperature. The western blots were developed using chemoluminescence and quantified by scanning densitometry .

***DNA isolation and real-time PCR***

The total DNA was extracted using a kit (QIAamp DNA Mini kit; Qiagen, Germany), and quantitative PCR was performed using 18S rRNA primers for a nuclear target sequence and primers for a mitochondrial DNA d-loop target. The following primers were used: mitochondrial d-loop forward, 5′-AATCTACCATCCTCCGTG-3′, and reverse, 5′-GACTAATGATTCTTCACCGT; 18S rRNA forward, 5′-CATTCGAACGTCTGCCCTATC-3′, and reverse: 5′-CCTGCTGCCTTCCTTGGA-3′. Quantitative PCR was performed using the Mx3000P Real-Time PCR system (Stratagene, La Jolla, CA, USA). The ratio of the mitochondrial d-loop to 18S was then calculated. The results are presented as fold increases over the control.

***RNA isolation and real-time PCR (RT-PCR)***

The total RNA was extracted using the TRIzol reagent (Invitrogen, Carlsbad, CA, USA) according to the manufacturer's instructions. The following primers were used: Nrf1 forward, 5′-GCCGTCGGAGCACTTACT-3’, and reverse, 5′-CTGTTCCAATGTCACCACC-3’; PGC1α forward, 5′-ATCTACTGCCTGGGGACCTT-3′, and reverse, 5′-ATGTGTCGCCTTCTTGCTCT-3’; Tfam forward, 5-CGCAGCACCTTTGGAGAA-3’, and reverse, 5′-CCCGACCTGTGGAATACTT-3′; 18S rRNA forward, 5′-CATTCGAACGTCTGCCCTATC-3′, and reverse, 5′-CCTGCTGCC TTCCTTGGA-3′. Quantitative PCR was performed using the Mx3000P real-time PCR system. Each quantitative PCR was performed in triplicate. The evaluation of the relative differences in the PCR product amounts among the treatment groups was performed using the ΔΔCT method. The reciprocal of 2CT for each target gene was normalized to that for 18S rRNA and then compared with the relative value in control cells .

***Statistical analysis***

All of the quantitative data are representative of at least three independent experiments. The data are presented as the means ± SEM. The statistical significance between the groups was determined by one-way ANOVA with Bonferroni’s test. The criterion for significance was set at *p*<0.05.

References

1. **Trounce IA, Kim YL, Jun AS, et al.** Assessment of mitochondrial oxidative phosphorylation in patient muscle biopsies, lymphoblasts, and transmitochondrial cell lines. *Methods Enzymol*.1996;**264**:484-509.

2. **Zheng J, Ramirez VD**. Inhibition of mitochondrial proton F0F1-ATPase/ATP synthase by polyphenolic phytochemicals. *Br J Pharmacol*.2000;**130**:1115-23.

3. **Picklo MJ, Montine TJ**. Acrolein inhibits respiration in isolated brain mitochondria. *Biochim Biophys Acta*.2001;**1535**:145-52.

4. **Addison PD, Neligan PC, Ashrafpour H, et al.** Noninvasive remote ischemic preconditioning for global protection of skeletal muscle against infarction. *Am J Physiol Heart Circ Physiol*.2003;**285**:H1435-43.

5. **Hao J, Shen W, Tian C, et al.** Mitochondrial nutrients improve immune dysfunction in the type 2 diabetic Goto-Kakizaki rats. *J Cell Mol Med*.2009;**13**:701-11.

6. **Shanker G, Aschner JL, Syversen T, et al.** Free radical formation in cerebral cortical astrocytes in culture induced by methylmercury. *Brain Res Mol Brain Res*.2004;**128**:48-57.

7. **Befroy DE, Petersen KF, Dufour S, et al.** Impaired mitochondrial substrate oxidation in muscle of insulin-resistant offspring of type 2 diabetic patients. *Diabetes*.2007;**56**:1376-81.

8. **Shen W, Liu K, Tian C, et al.** R-alpha-lipoic acid and acetyl-L-carnitine complementarily promote mitochondrial biogenesis in murine 3T3-L1 adipocytes. *Diabetologia*.2008;**51**:165-74.

9. **Hayakawa T, Noda M, Yasuda K, et al.** Ethidium bromide-induced inhibition of mitochondrial gene transcription suppresses glucose-stimulated insulin release in the mouse pancreatic beta-cell line betaHC9. *J Biol Chem*.1998;**273**:20300-7.

10. **Shen W, Hao J, Tian C, et al.** A combination of nutriments improves mitochondrial biogenesis and function in skeletal muscle of type 2 diabetic Goto-Kakizaki rats. *PLoS ONE*.2008;**3**:e2328.
