# Supplementary figures and images for: Chemerin-induced mitochondrial dysfunction in skeletal muscle
Source: J Cell Mol Med. 2015 Mar 6;19(5):986–95. doi: 10.1111/jcmm.12487 (PMC4420601; doi:10.1111/jcmm.12487)

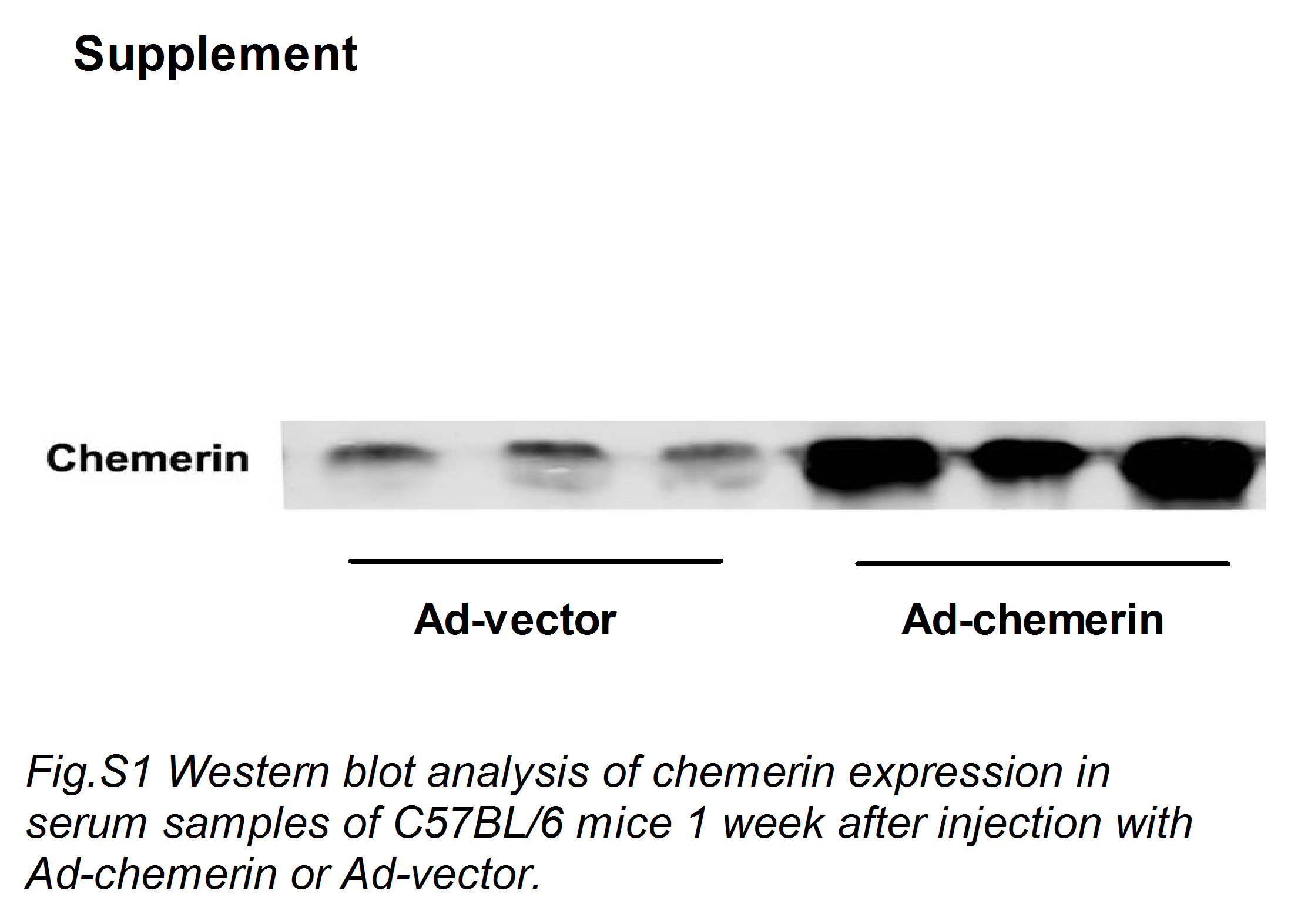

Supplement: Supplementary file 2 [file jcmm0019-0986-sd2.tif]
